# Supplementary material for: MT4-MMP deficiency increases patrolling monocyte recruitment to early lesions and accelerates atherosclerosis
Source: Nat Commun. 2018 Mar 2;9:910. doi: 10.1038/s41467-018-03351-4 (PMC5834547; doi:10.1038/s41467-018-03351-4)
Supplement: Supplementary file 1 — Supplementary Information [file 41467_2018_3351_MOESM1_ESM.pdf]

SUPPLEMENTARY INFORMATION

MT4-MMP DEFICIENCY INCREASES PATROLLING MONOCYTE RECRUITMENT TO EARLY LESIONS AND ACCELERATES ATHEROSCLEROSIS

Cristina Clemente, Cristina Rius, Laura Alonso-Herranz, Mara Martín-Alonso, Ángela Pollán, Emilio Camafeita, Fernando Martínez, Rubén A. Mota, Vanessa Núñez, Cristina Rodríguez, Motoharu Seiki, José Martínez-González, Vicente Andrés, Mercedes Ricote, and Alicia G. Arroyo*

* Correspondence to: agarroyo@cnic.es

SUPPLEMENTARY FIGURES

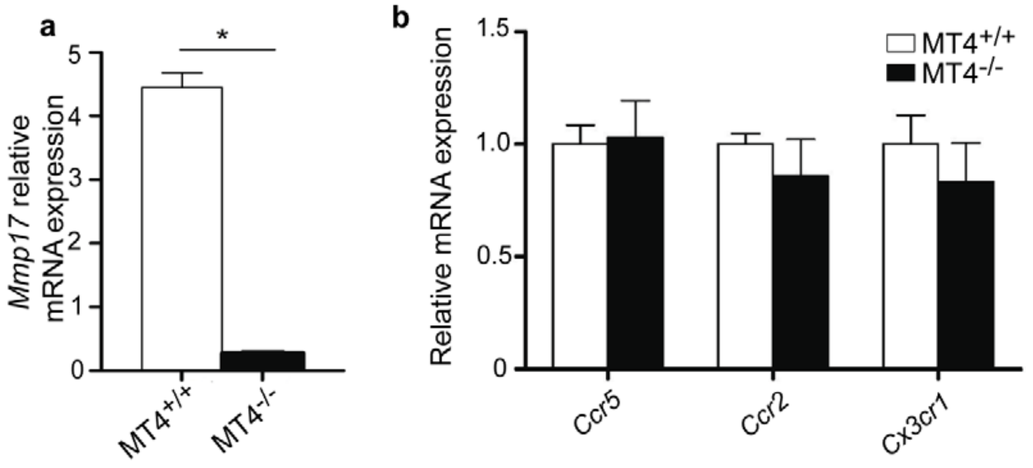

Supplementary Fig. 1. MT4-MMP is expressed in TG-elicited peritoneal macrophages. qPCR analysis of (**a**) *Mmp17* (MT4-MMP) and (**b**) *Ccr5*, *Ccr2*, *Cx3cr1* mRNA levels in TG-elicited macrophages from wild-type and MT4-MMP-null mice adhered to plastic overnight; n= 6 per genotype in 2 independent experiments. Data were tested by two-tailed Student's t-test. Results are expressed as mean ± SEM. *p<0.05.

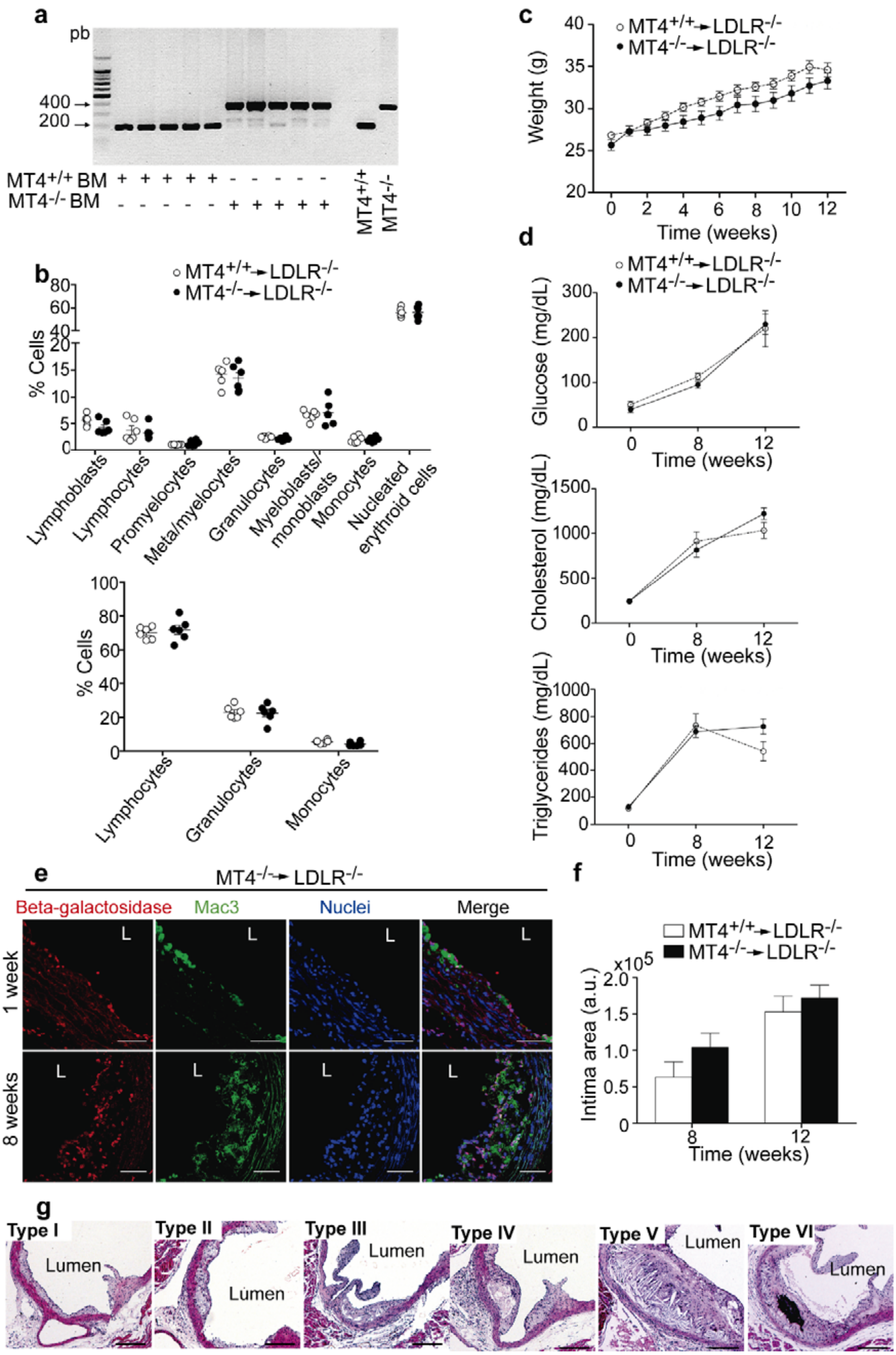
a
pb
400
200
MT4+/+ BM
MT4-/- BM
MT4+/+
MT4-/-
b
MT4+/+→LDLR-/-
MT4-/-→LDLR-/-
% Cells
Lymphoblasts
Lymphocytes
Promyelocytes
Meta/myelocytes
Granulocytes
Myeloblasts/monoblasts
Monocytes
Nucleated erythroid cells
Lymphocytes
Granulocytes
Monocytes
c
Weight (g)
Time (weeks)
d
Glucose (mg/dL)
Cholesterol (mg/dL)
Triglycerides (mg/dL)
e
MT4-/-→ LDLR-/-
Beta-galactosidase
Mac3
Nuclei
Merge
1 week
8 weeks
f
Intima area (a.u.)
x10^5
g
Type I
Type II
Type III
Type IV
Type V
Type VI
Lumen

Supplementary Fig. 2. AT-related parameters in *Ldlr*-deficient mice transplanted with MT4-MMP-null BM cells. (**a**) Engraftment analyzed by PCR of genomic DNA from blood cells of BM-transplanted *Ldlr*$^{-/-}$ mice. (**b**) Bone marrow (upper) and blood (lower) myeloid populations of BM-transplanted *Ldlr*$^{-/-}$ mice after engraftment. n=6 mice per genotype in 2 independent experiments. (**c**) Body weight and (**d**) serum glucose, triglyceride, and total cholesterol in BM-transplanted *Ldlr*$^{-/-}$ mice at different times after HFD feeding; n=6 and n=16 mice per genotype for 8 and 12 weeks in 2 and 3 independent experiments, respectively. (**e**) Representative microscopy images of transverse aortic sinus sections stained with anti-beta-galactosidase (red), anti-Mac3 (green), and Hoechst (blue, nuclei) in MT4-MMP$^{-/-}$-transplanted *Ldlr*$^{-/-}$ mice fed a HFD for 1 or 8 weeks; scale bar, 50 µm. (**f**) Intima area in the aortic sinus of BM-transplanted *Ldlr*$^{-/-}$ mice fed a HFD for 8 or 12 weeks; n=6 and n=16 mice per genotype for 8 and 12 weeks in 2 and 3 independent experiments, respectively. (**g**) Representative images of H&E-stained transverse sections of aortic sinus from BM-transplanted *Ldlr*$^{-/-}$ mice fed a HFD and scored on the Stary classification from I to VI (Stary et al., 1995); scale bar, 200 µm. Data were tested by Student´s t-test in (b) and by two-way ANOVA followed by Bonferroni's post-test in (**c**), (**d**) and (**f**). Results are expressed as mean ± SEM.

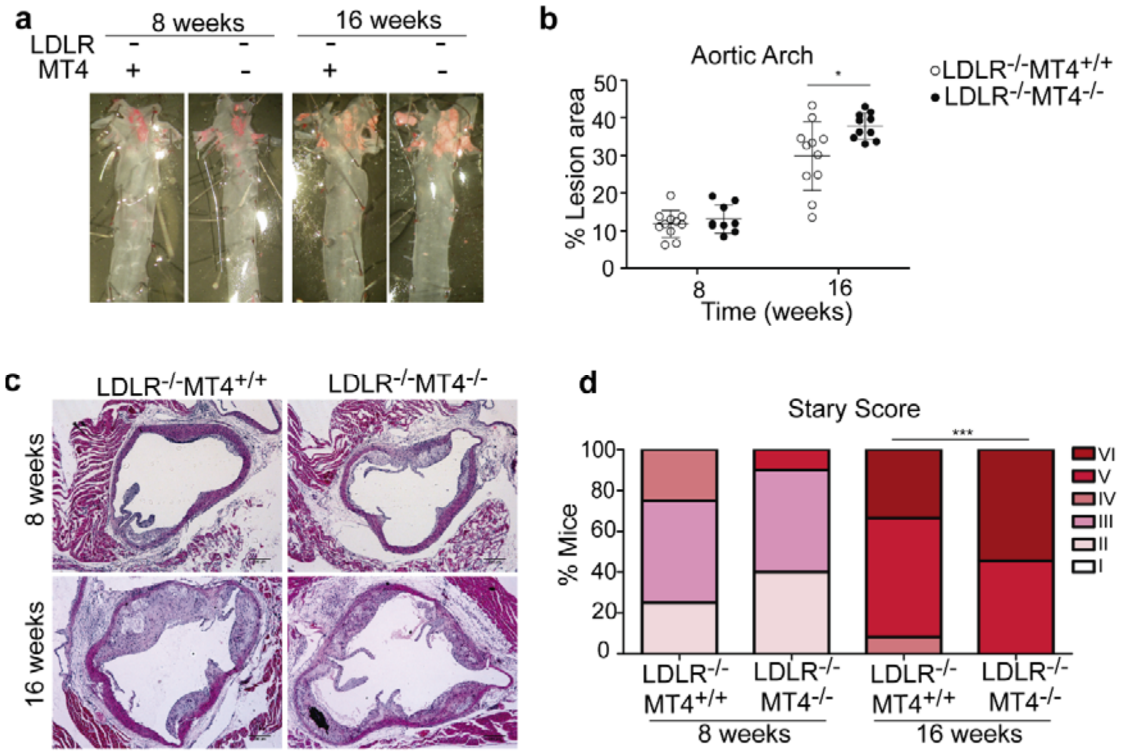

Supplementary Fig. 3. Accelerated atherosclerosis in MT4-MMP$^{-/-}$ *Ldlr*$^{-/-}$ double-deficient mice mice fed a HFD. (**a**) Representative images of en face Oil-Red staining in aortas from *Ldlr*$^{-/-}$ mice or *Ldlr*$^{-/-}$/MT4-MMP$^{-/-}$ double-deficient mice fed a HFD for 8 or 16 weeks. (**b**) Oil-Red-positive lesion area (%) in the aortic arch; n=8-12 mice per genotype and time-point. (**c**) Representative H&E-stained transverse sections from aortic sinus of *Ldlr*$^{-/-}$ and double-deficient mice fed a HFD for 8 and 16 weeks; scale bar, 200 µm. (**d**) Stary scoring (I-VI) in *Ldlr*$^{-/-}$ and *Ldlr*$^{-/-}$/MT4-MMP$^{-/-}$ mice, shown as a percentage of all mice for each condition after feeding a HFD for 8 or 16 weeks; n=8-12 mice per genotype and time-point. Data were tested by two-way ANOVA followed by Bonferroni's post-test in (**b**) and by Chi-square test for a trend in (**d**). Results are expressed as mean ± SEM. *p<0.05 and ***p<0.001.

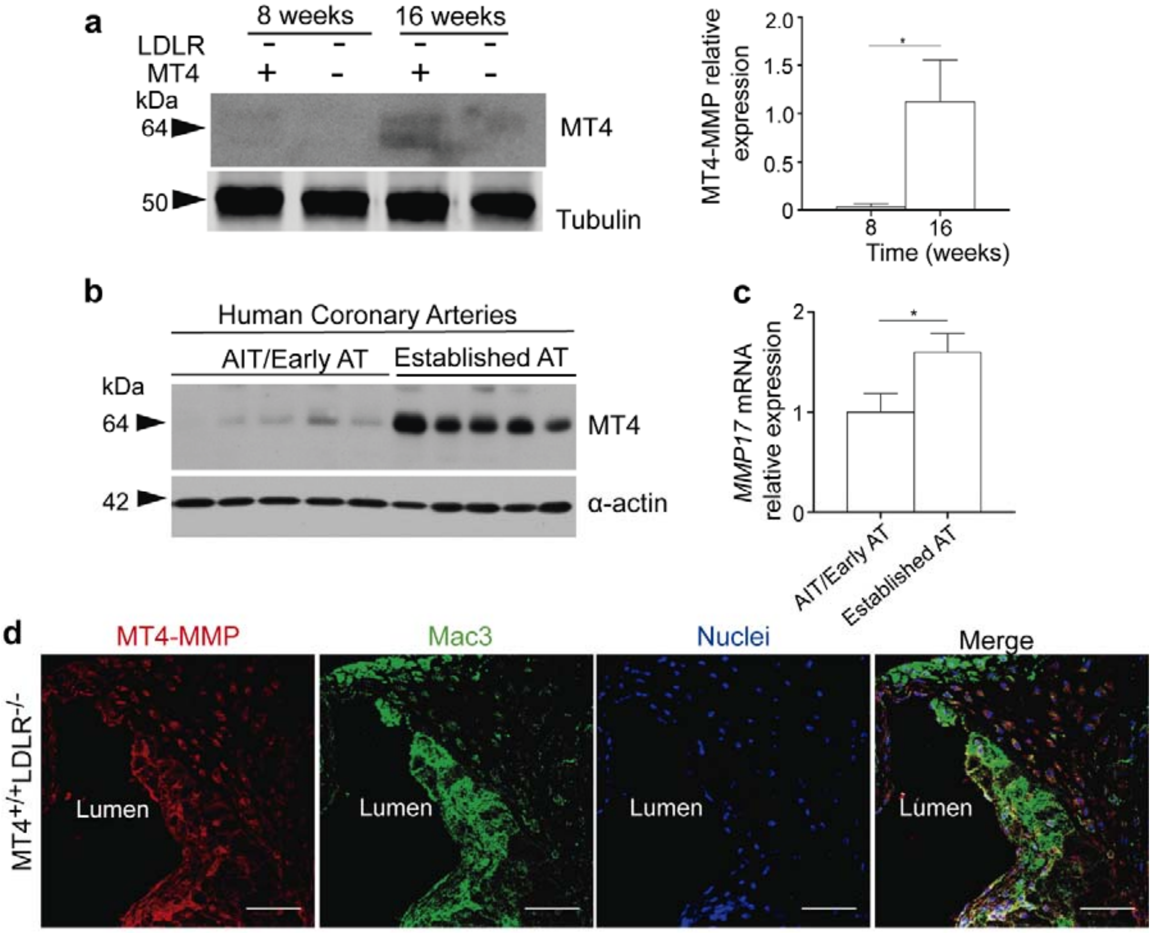

Supplementary Fig. 4. MT4-MMP is expressed by macrophages in atherosclerotic plaques. (**a**) Representative western blot analysis of MT4-MMP expression in protein extracts from paraffin-embedded sections of aortas from *Ldlr*$^{-/-}$/MT4-MMP$^{+/+}$ and *Ldlr*$^{-/-}$/MT4-MMP$^{-/-}$ mice fed a HFD for 8 or 16 weeks. Tubulin is included as a loading control; quantification of MT4-MMP from the *Ldlr*$^{-/-}$/MT4$^{+/+}$ samples is shown on the right; n= 4 mice per genotype and time-point. (**b**) Western blot of MT4-MMP protein in human coronary arteries. (**c**) qPCR analysis of *Mmp17* (MT4-MMP) mRNA in human coronary arteries. Coronary artery samples showed arterial intima thickening (AIT) [n=10] or established AT [n=15]). (**d**) Representative microscopy images of transverse aortic sinus sections stained with anti-MT4-MMP (red), ant-Mac3+ (green), and Hoechst (nuclei, blue) in *Ldlr*$^{-/-}$/MT4-MMP$^{+/+}$ mice fed a HFD for 8 weeks; scale bar, 50 µm. Data were tested by Student's t-test. Results are expressed as mean ± SEM. *p<0.05

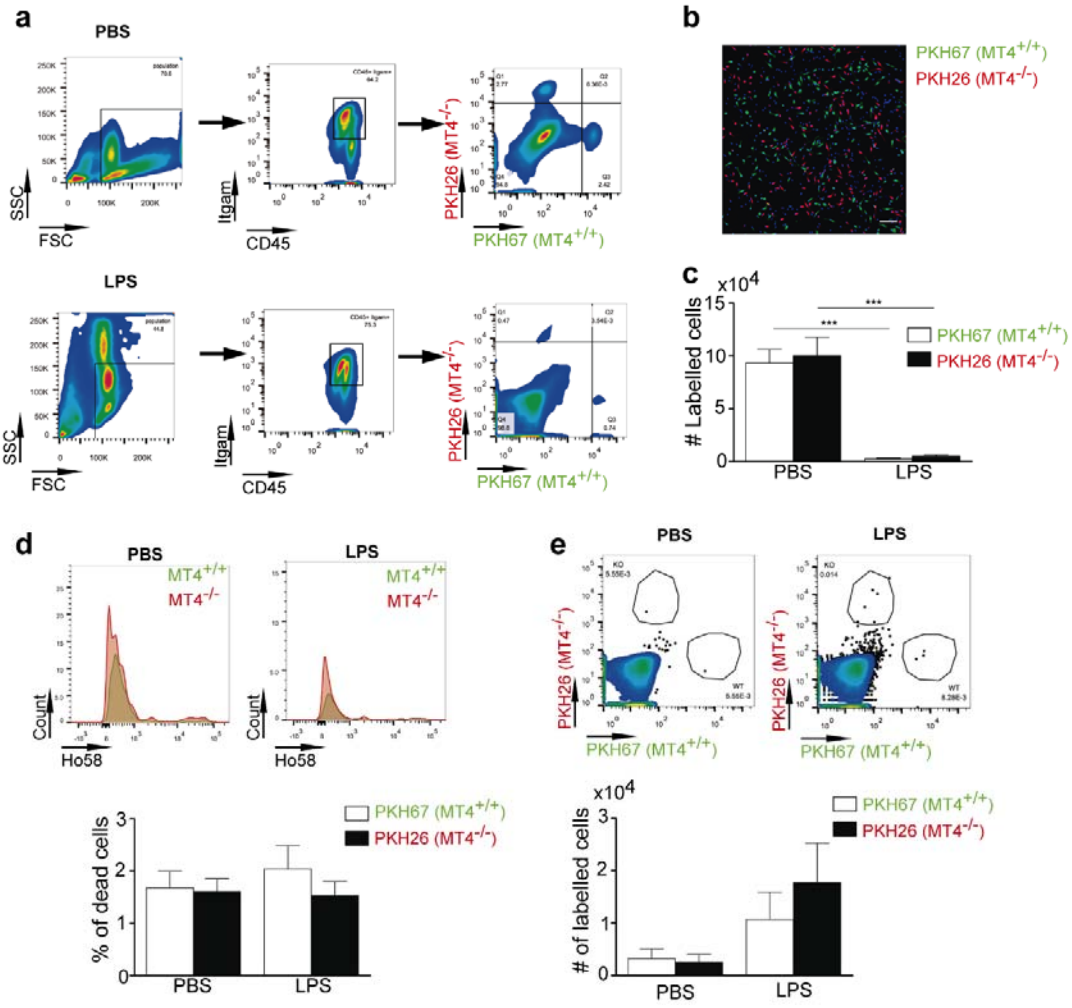

Supplementary Fig. 5. MT4-MMP absence does not affect macrophage egression from inflamed tissues. (**a**) Representative flow cytometry dot plots of the peritoneal lavage from wild-type mice stimulated with thioglycollate for 72 hours, injected i.p. with dual-labeled wild-type (PKH67, green) and MT4-MMP-null (PKH26, red) macrophages, and analyzed after 4 hours of PBS or LPS stimulation (1µg i.p.). (**b**) Representative microscopy image of TG-elicited macrophages labelled as in a seeded overnight on a glass coverslip. Scale bar, 100 µm. (**c**) Graph shows the quantification of the number of labelled peritoneal cells gated in a. (**d**) Representative flow cytometry histograms of Hoechst 33258 (Ho58) staining (top) and graph showing the percentage of dead (Ho58+) cells (bottom) within the populations gated in a. (**e**) Representative flow cytometry dot plots of spleen cells from mice analyzed in a (top) and graph showing the number of labelled cells recovered (bottom). n=6 mice per genotype and treatment in 2 independent experiments. Statistical analysis was performed using one-way ANOVA followed by Bonferroni´s post-test. Results are expressed as mean ± SEM. $*p < 0.05$, $**p < 0.01$, $***p<0.001$

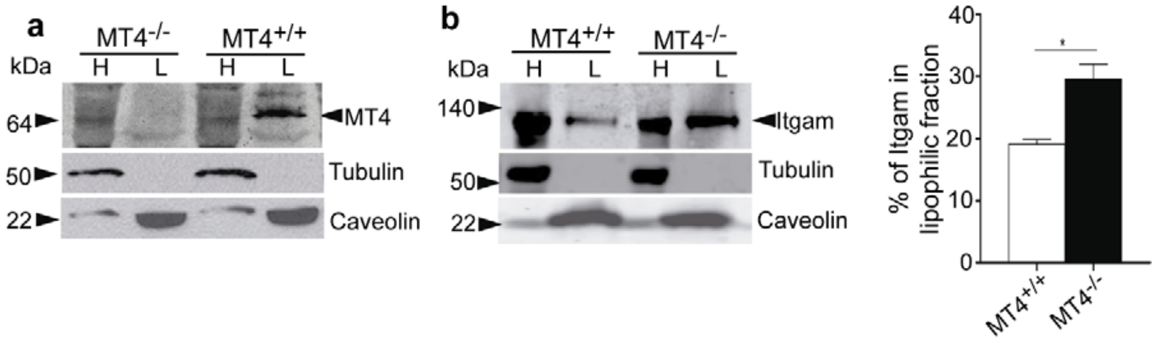

Supplementary Fig. 6. MT4-MMP and Itgam are expressed in lipid-rich domains from TG-elicited peritoneal macrophages. (**a**) Representative western blot for MT4-MMP of TX-114 lysates from wild-type ($MT4^{+/+}$) and $MT4\text{-}MMP^{-/-}$ ($MT4^{-/-}$) 72 h TG-elicited macrophages adhered to plastic overnight. (**b**) Representative western blot for Itgam of TX-114 lysates from $MT4^{+/+}$ and $MT4^{-/-}$ 72 h TG-elicited macrophages (left) and quantification of Itgam levels in the lipophilic fraction (right); n=6 samples per genotype; H= hydrophilic and L=lipophilic fractions. Tubulin and caveolin-1 are included as controls for the hydrophilic and lipophilic fractions, respectively. Data were tested by Student's t-test. Results are expressed as mean ± SEM. *$p<0.05$.

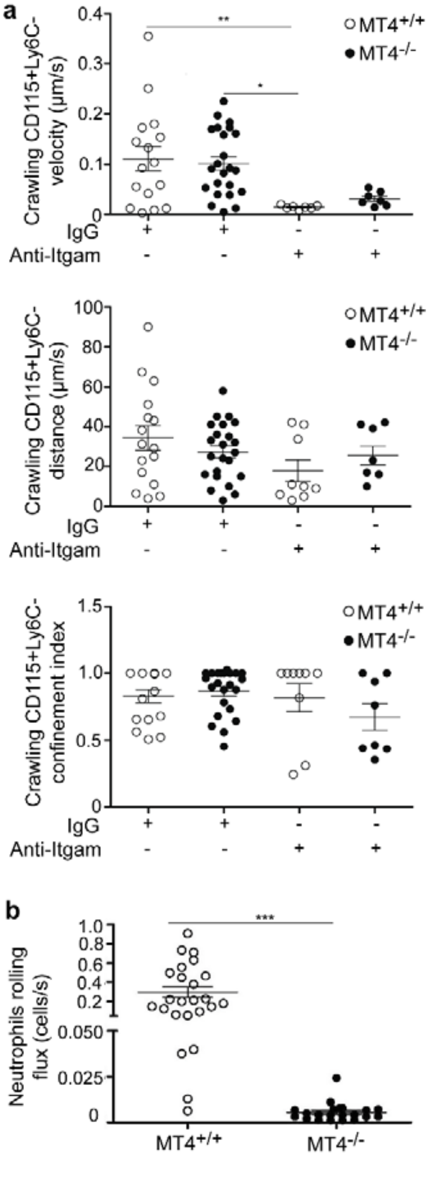

Supplementary Fig. 7. Patrolling monocyte behavior in the absence of MT4-MMP. (**a**) Crawling velocity, total distance traveled, and confinement index (lineal distance/total distance traveled) of CD115+Ly6C- monocytes in MT4-MMP$^{+/+}$ (MT4$^{+/+}$) and MT4-MMP$^{-/-}$ (MT4$^{-/-}$) mice analyzed by intravital microscopy in CCL2-inflamed cremaster muscle in the presence of the ligand-blocking anti-Itgam antibody M1/70 or it corresponding IgG isotype control; it is represented every quantified cell from 5 mice per genotype and condition. (**b**) Neutrophil (CD115+Ly6G+) rolling in MT4$^{+/+}$ and MT4$^{-/-}$ mice analyzed by intravital microscopy in the CCL2-inflamed cremaster muscle. Values are presented from every quantified vein in 8 independent mice per genotype. Data were tested by one-way ANOVA followed by Bonferroni's post-test in (**a**) and by Student's t-test in (**b**). Results are expressed as mean ± SEM. ***$p<0.001$.

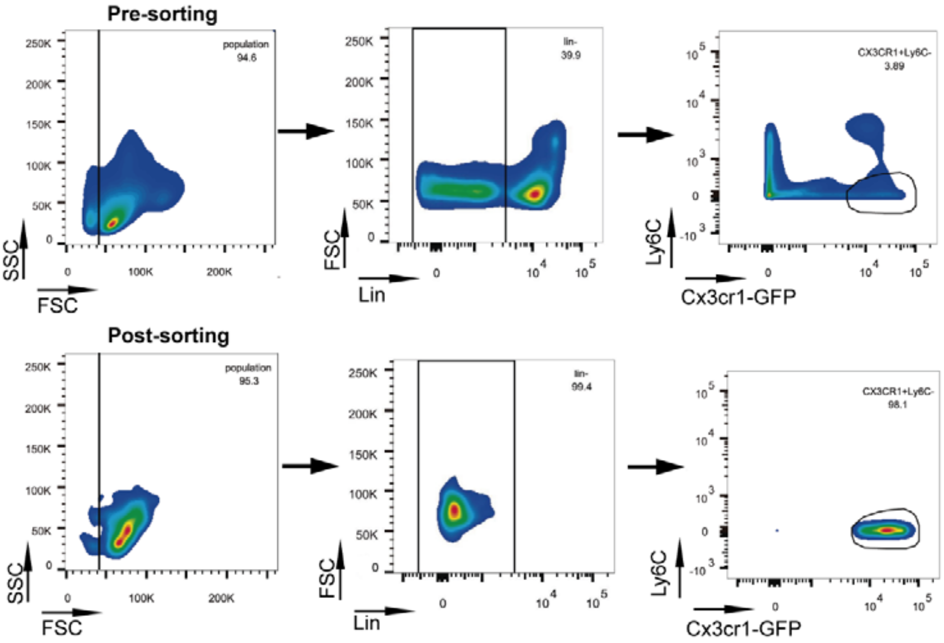

Supplementary Fig. 8. Strategy for patrolling monocyte sorting from the spleen of *Cx3cr1*$^{Gfp/+}$ reporter mice. Spleen cells were gated by their size (FSC) and complexity (SSC), selected by the negative expression of lineage (Lin) markers (CD3, B220, Ly6G and NK1.1) and patrolling monocytes finally sorted as Lin-negative, Cx3cr1-GFP-positive and Ly6C-negative cells. Representative pre-sorting and post-sorting flow cytometry dot plots of the spleen cells from *Cx3cr1*$^{Gfp/+}$ mice are shown.

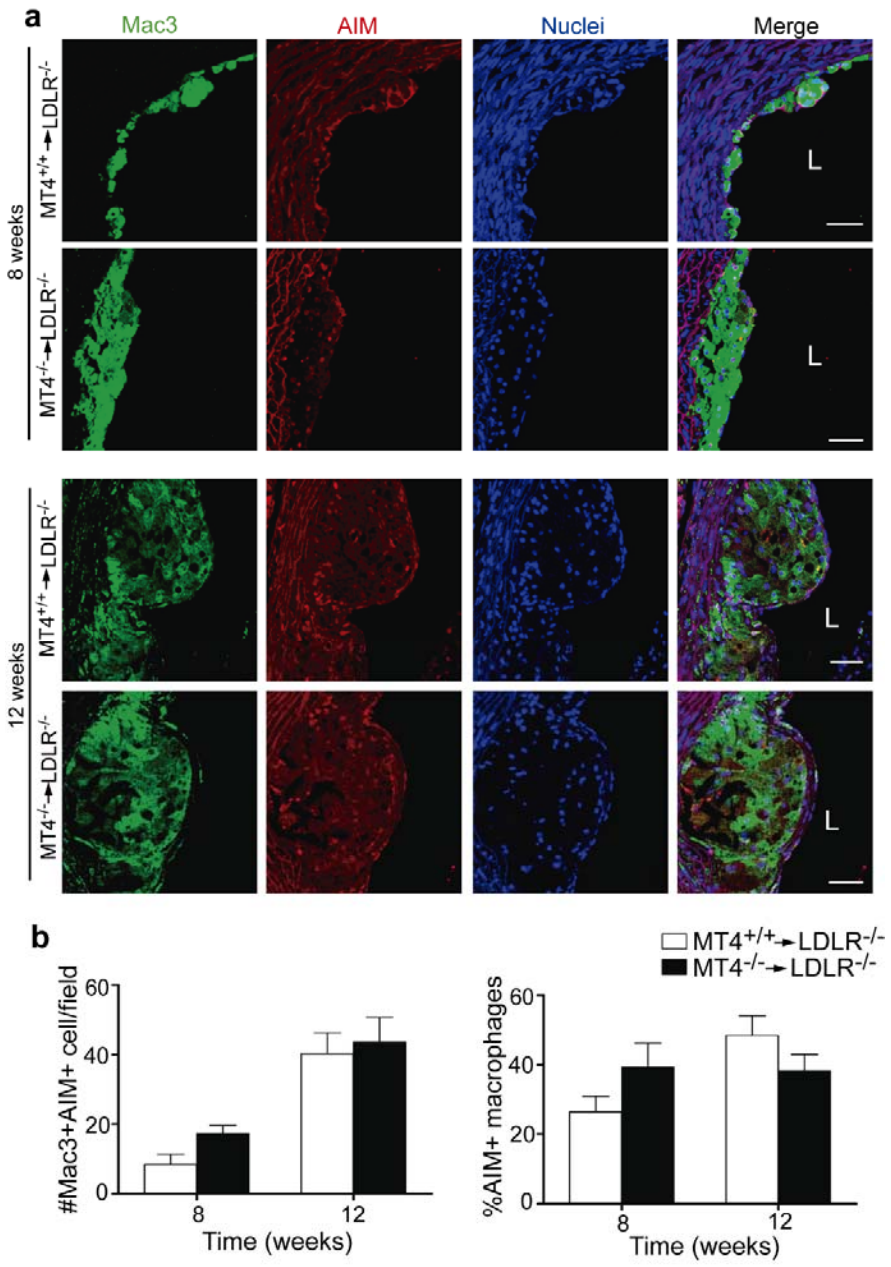

Supplementary Fig. 9. AIM expression in macrophages in advanced AT plaques in the absence of MT4-MMP. (**a**) Representative confocal microscopy images of transverse sections from the aortic sinus of *Ldlr*$^{-/-}$ mice transplanted with MT4$^{+/+}$ or MT4$^{-/-}$ bone marrow cells and fed a HFD for 8 or 12 weeks. Mac-3 (green), AIM (red), and Hoechst (blue, nuclei). Scale bar 50µm. L indicates the lumen. (**b**), Bar graph shows the quantification of the number of Mac-3+AIM+ macrophages (left) and the percentage of AIM+ macrophages (right). n=5 mice per genotype and time point in 2 independent experiments. Data were tested by two-way ANOVA followed by Bonferroni's post-test. Results are expressed as mean ± SEM.

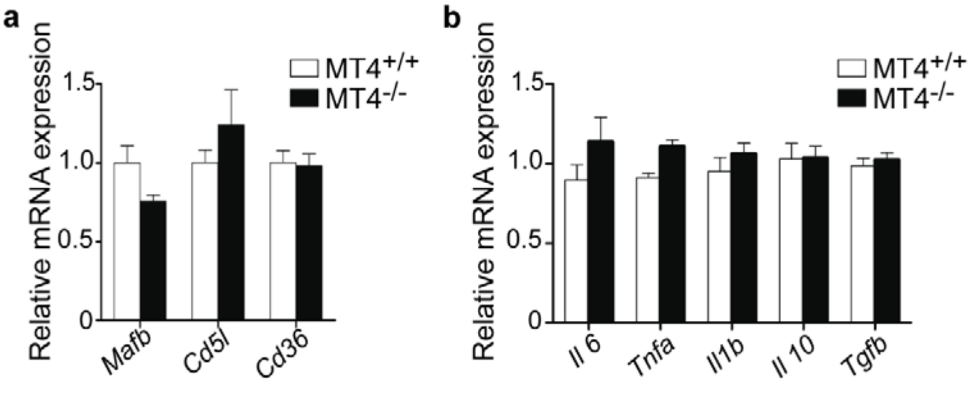

Supplementary Fig. 10. Profiling of MT4-MMP-null peritoneal macrophages. qPCR analysis of (**a**) *Mafb*, *Cd5l* (Aim), and *Cd36* and (**b**) proinflammatory cytokines (*Il6*, *Tnfa* and *Il1b*) and antiinflammatory cytokines (*Il10* and *Tgfb*) in peritoneal macrophages from MT4-MMP$^{+/+}$ (MT4$^{+/+}$) and MT4-MMP$^{-/-}$ (MT4$^{-/-}$) mice obtained 72 hours after TG stimulation and adhered to plastic dishes overnight; n=6 mice per genotype in 2 independent experiments. Data were analyzed by two-tailed Student's t-test. Results are expressed as mean ± SEM.

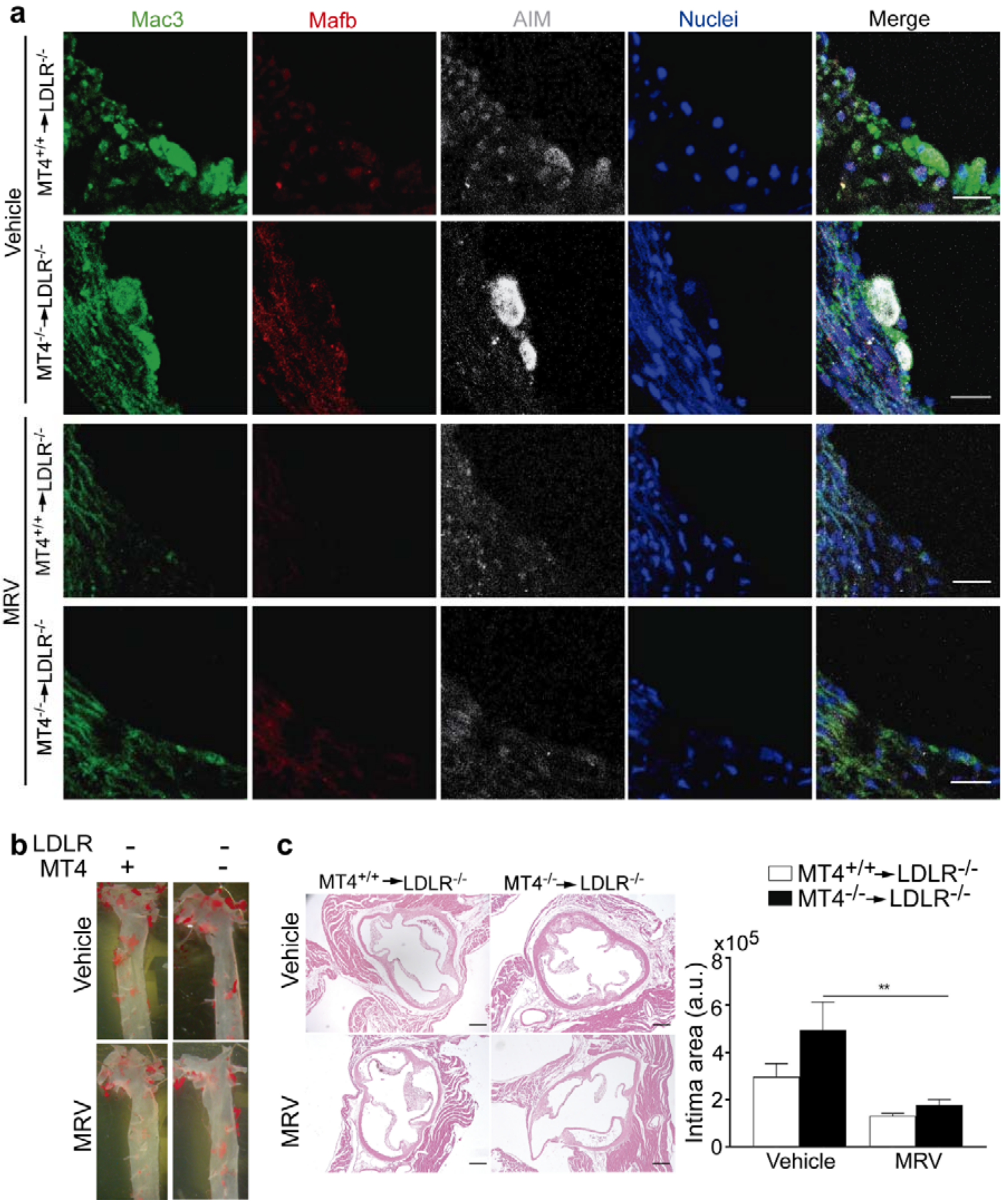

Supplementary Fig. 11. CCR5 inhibition hinders the AT phenotype in MT4-MMP-null BM-transplanted *Ldlr*$^{-/-}$ mice. (**a**) Representative images of transverse sections of aortic sinus from *Ldlr*$^{-/-}$ mice transplanted with MT4-MMP$^{+/+}$ (MT4$^{+/+}$) or MT4-MMP$^{-/-}$ (MT4$^{-/-}$) BM cells and fed a HFD for 7 days and treated with Maraviroc (MRV, 25µg/g every 12 hours) or vehicle. n=7 and n=8 mice for Vehicle and MRV mice per genotype in 2 independent experiments; sections were labeled for Mac3 (green), Mafb (red), AIM (white), and Hoechst (blue, nuclei); scale bar, 20 µm. (**b**) Representative images of en face Oil Red-

stained aortas from BM-transplanted *Ldlr*$^{-/-}$ mice fed a HFD for 8 weeks and treated with Maraviroc or vehicle. n=7 and n=8 mice for Vehicle and MRV mice per genotype in 2 independent experiments. (**c**) Representative images of aortic sinus stained with H&E from BM-transplanted *Ldlr*$^{-/-}$ mice fed a HFD for 8 weeks and treated with Maraviroc or vehicle (left; scale bar, 200 µm) and quantification of intima area (right). n=7 and n=8 mice for Vehicle and MRV mice per genotype in 2 independent experiments. Data were tested by one-way ANOVA followed by Bonferroni´s post-test. Results are expressed as mean ± SEM. *p<0,05, ***p<0,005.

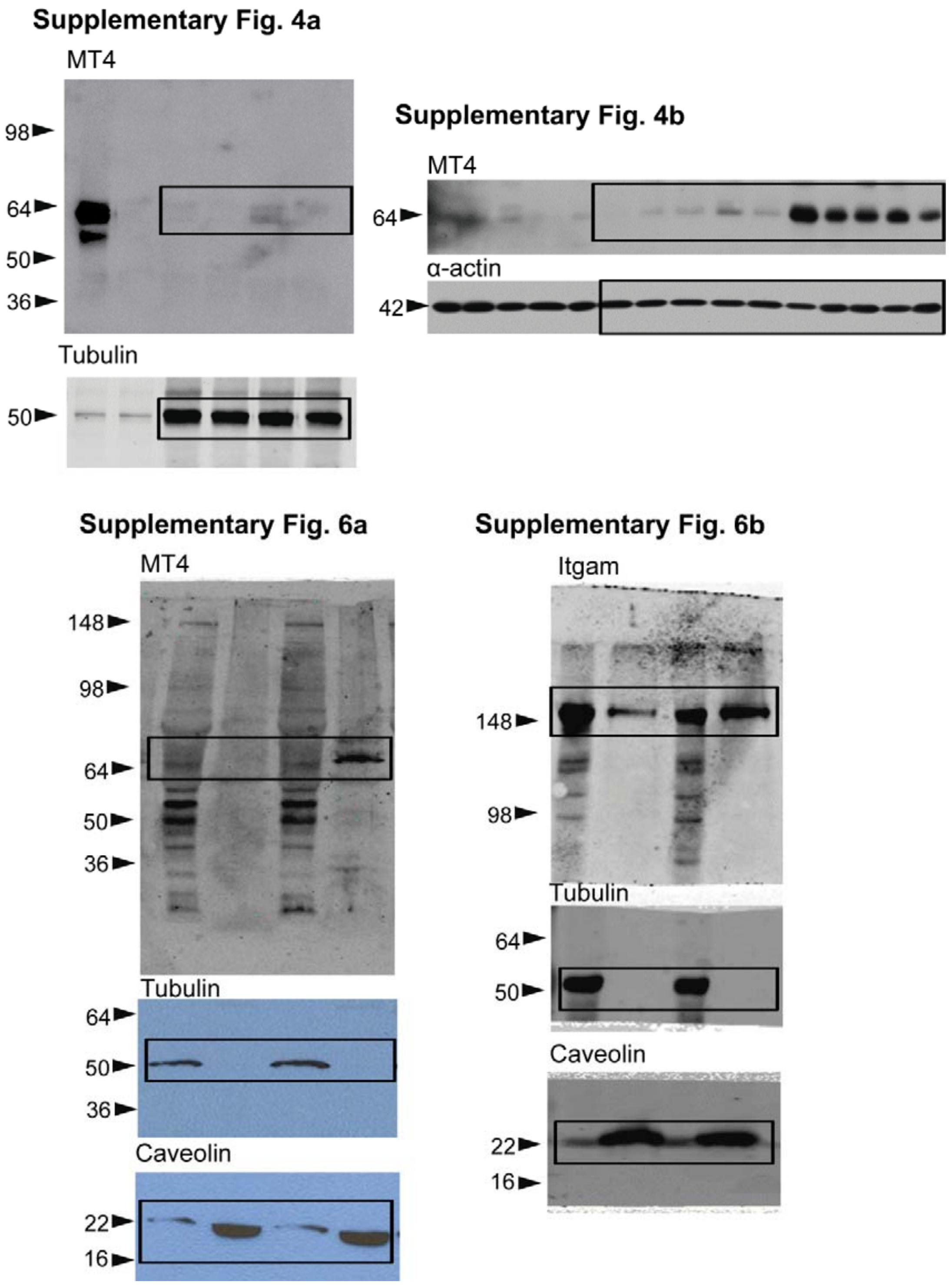

Supplementary Fig. 12. Uncropped western blots used for the Supplementary Figures.

SUPPLEMENTARY TABLES

Supplementary Table 1. qPCR primers used in this study

Gene	Direction	Primer Sequence
**Itgam**	Forward	5´-GCTGAAAGCTCTCCACCTCA-3´
	Reverse	5´-AGGCCACAGGTATTTTGTCG-3´
**Mmp17**	Forward	5´-ACTGTCCAAAGCGATTACTGC-3´
	Reverse	5´-GCATCGAGGGGTTTTCATCAG-3´
**Il6**	Forward	5´-TAGTCCTTCCTACCCCAATTTCC-3´
	Reverse	5´-TTGGTCCTTAGCCACTCCTTC-3´
**Tnfa**	Forward	5´-CCAGACCCTCACACTCAGATC-3´
	Reverse	5´-CACTTGGTGGTTTGCTACGAC-3´
**Il1b**	Forward	5´-GCTGAAAGCTCTCCACCTCA-3´
	Reverse	5´-AGGCCACAGGTATTTTGTCG-3´
**Il10**	Forward	5´-TGAATTCCCTGGGTGAGAAG-3´
	Reverse	5´-TCACTCTTCACCTGCTCCACT-3´
**Tgfb**	Forward	5´-GCTAATGGTGGACCGCAAC-3´
	Reverse	5´-CGAATGTCTGACGTATTGAAGAA-3´
**Mafb**	Forward	5´-AGGACCGCTTCTCTGATGA-3´
	Reverse	5´-GAGCTGCGTCTTCTCGTTCT-3´
**Cd5l**	Forward	5´-GGAAGACACGTTGGCTCAAT-3´
	Reverse	5´-AGACGCACATCCTCTGGAAT-3´
**Cd36**	Forward	5´-GAGCAACTGGTGGATGGTTT-3´
	Reverse	5´-GCAGAATCAAGGGAGAGCAC-3´
**Ccr5**	Forward	5´-TGCAGTCCTCATTTTCCACA-3´
	Reverse	5´-CCTACAGCGAAACAGGGTGT-3´
**Ccr2**	Forward	5´-ACACCCTGTTTCGCTGTAGG-3´
	Reverse	5´-CCTGGAAGGTGGTCAAGAAG-3´
**Cx3Cr1**	Forward	5´-AAGTTCCCTTCCCATCTGCT-3´
	Reverse	5´-CGAGGACCACCAACAGATTT-3´
**Cyclophilin**	Forward	5´-ACAGGTCCTGGCATCTTGTC-3´
	Reverse	5´-CATGGCTTCCACAATGTTCA-3´
**36b4**	Forward	5´-GCGACCTGGAAGTCCAACTA-3´
	Reverse	5´-ATCTGCTGCATCTGCTTGG-3´
