# Supplementary material for: MT4-MMP deficiency increases patrolling monocyte recruitment to early lesions and accelerates atherosclerosis
Source: Nat Commun. 2018 Mar 2;9:910. doi: 10.1038/s41467-018-03351-4 (PMC5834547; doi:10.1038/s41467-018-03351-4)
Supplement: Supplementary file 3 — Description of Additional Supplementary Files [file 41467_2018_3351_MOESM3_ESM.pdf]

Description of Additional Supplementary Files

File Name: Supplementary Data 1
Description: **List of predicted cleavage sites in the human αMβ2 integrin by the protease MT4-MMP.** List of predicted cleavage sites in the human αMβ2 integrin by the protease MT4-MMP determined by CleavPredict (http://cleavpredict.sanfordburnham.org/). A and B after the residue number denotes its presence in the αM or β2 integrin chains. Those exposed sites with higher score for cleavage have been highlighted in grey. Cleavage site in position 977A was selected for validation (highlighted in green).

File Name: Supplementary Movie 1
Description: **Crawling of patrolling monocytes in MT4-MMP WT mice with IgG.** Directly labeled antibodies to CD115 (red) and Ly6C (green) were injected into mice previously injected with isotype IgG. Intravital microscopy recording in the cremaster was performed 4 h after injection of CCL2.

File Name: Supplementary Movie 2
Description: **Crawling of patrolling monocytes in MT4-MMP-null mice with IgG.** Directly labeled antibodies to CD115 (red) and Ly6C (green) were injected into mice previously injected with isotype IgG. Intravital microscopy recording in the cremaster was performed 4 h after injection of CCL2.

File Name: Supplementary Movie 3
Description: **Crawling of patrolling monocytes in MT4-MMP WT mice with Itgam blocking antibody.** Directly labeled antibodies to CD115 (red) and Ly6C (green) were injected into mice previously injected with anti-Itgam blocking antibody. Intravital microscopy recording in the cremaster was performed 4 h after injection of CCL2.

File Name: Supplementary Movie 4
Description: **Crawling of patrolling monocytes in MT4-MMP-null mice with Itgam blocking antibody.** Directly labeled antibodies to CD115 (red) and Ly6C (green) were injected into mice previously injected with anti-Itgam blocking antibody. Intravital microscopy recording in the cremaster was performed 4 h after injection of CCL2.
